# Compact coherent perfect absorbers using topological guided-mode resonances

**DOI:** 10.1038/s41598-024-63605-8

**Published:** 2024-06-19

**Authors:** Chan Young Park, Ki Young Lee, Yu Sung Choi, Jae Woong Yoon

**Affiliations:** 1 Department of Physics, Hanyang University, Seoul, 04763 Korea; 2 Research Institute for Natural Sciences, Hanyang University, Seoul, 04763 Korea

**Keywords:** Metamaterials, Nanophotonics and plasmonics

## Abstract

We propose a topological coherent perfect absorber that enables almost ideal performance with remarkably compact device footprint and tight incident beams. The proposed structure is based on a topological junction of two guided-mode-resonance gratings. The structure provides robust systematic ways of remarkably tight lateral confinement of the absorbing resonance mode and near-perfect mode-match to arbitrary incident beams, which are unavailable with the conventional approaches. We demonstrate an exemplary amorphous Si thin-film structure that enables near-perfect absorptance modulation between 1.7 and 99% with device footprint width of 30-μm and 10-μm-wide incident Gaussian beams. Therefore, our proposed approach greatly improves practicality of guided-mode-resonance coherent perfect absorbers.

## Introduction

In a lossy scattering system, complete annihilation of electromagnetic radiation fields is possible by appropriately designing structure geometry and coherent incident-waveform configuration. Such devices are known as coherent perfect absorbers^1,2^ They in general require resonant elements such as optical cavities, waveguides, and nanoparticles^3^. When such elements are configured at a certain optimal condition referred to as the critical coupling condition^4^, it enables remarkably efficient phase-sensitive optical modulation between two extreme states—the coherent perfect absorption (CPA) and coherent total scattering (CTS)^5^. Various CPA devices have been proposed on the bases of thin films^6^, graphene nanostructures^7^, metallic gratings^8^, and dielectric metasurfaces^9^.

Toward this end, guided-mode resonance (GMR) absorbers have been studied because of their technical advantages including structural simplicity, compactness, almost freely adjustable operation band and Q factor, polarization selectivity, and many others^10,11^. There are fundamental limitations of GMR CPA devices. Conventional GMR CPA devices demand extremely uniform nanostructures over a considerably large surface area (~ 1 mm^2^) in order to secure desired performance^12,13^. This originates from narrow angular tolerance of GMRs, associated diffraction-limited beam diameters, and in-plane delocalization of resonant modes as bulk Bloch-Floquet states in principle^14,15^. Therefore, significant degradation of device performance is inevitable if tight incident beams or small-footprint devices are required in favor of their practical applications.

In this paper, we propose a GMR CPA-device structure based on a diffractive topological junction as an efficient solution to this problem. We utilize a photonic Jackiw–Rebbi (JR) state at a junction of two topologically distinguished GMR gratings^16,17^. Under 10-μm-wide Gaussian beam incidence on an optimized amorphous-Si (α-Si) device, we numerically demonstrate a phase-sensitive switching between the CPA and CTS states with their absorptance values at 99% and 1.7%, respectively. This remarkably high performance is enabled by tight lateral confinement of the GMR JR state and a unique mode-matching capability to the incident beams by means of adoptable Dirac-mass shaping. Importantly, such high performance is crucial for practical applications such as coherence filters, modulators, and pulse recovery^18^.

## Results

### Impact of finite beam and device sizes

**Figure 1:**
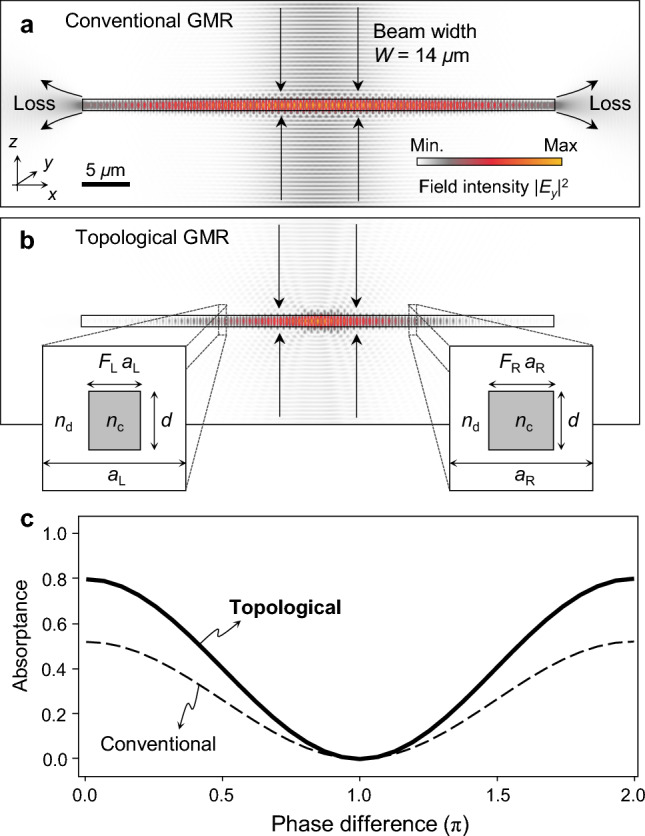
Basic concept of a topological GMR CPA. (**a**,**b**) Electric field intensity *|E*_*y*_*|*^2^ distributions in the CPA operation for conventional and topological GMRs with footprint size 50 μm, Gaussian beam width *W* = 14 μm under normal incidence of TE-polarized light at wavelengths 1468 and 1493 nm, respectively. (**c**) Enhanced phase-sensitive absorptance-modulation profile of the topological GMR CPA device in comparison with the conventional GMR at the critical coupling condition.

We first consider a conventional GMR CPA device in order to describe our proposed approach in comparison. In Fig. 1a, we show a conventional GMR CPA device and its resulting field distribution, respectively. We assume thickness *d* = 500 nm, core refractive index *n*_c_ = 3.485 (α-Si), clad refractive index *n*_d_ = 2.45 (Si_3_N_4_), period *a* = 540 nm, and fill factor *F* = 0.45 in this calculation. In addition, we include intrinsic loss of α-Si at 0.0052, which is reasonable from experimental data in the literature^19,20^. We use the finite element method (FEM) for the numerical analyses. See Method section for details of the simulation conditions.

This trial device induces a critically-coupled GMR at wavelength 1468 nm under normal incidence of transverse-electric (TE)-polarized light and thereby it can function as a CPA device under planewave incidence in principle. For two coherent Gaussian-beam incidences with beam width 14 μm, an incomplete CPA with its absorptance degraded down to ~ 50% is obtained. The imperfection is significant and originates in two major factors—in-plane leakage losses and modal mismatch between the incident field and resonance mode. In a fundamental viewpoint, these two factors are inevitable because GMR states are basically delocalized Bloch–Floquet states with its coupled radiation modes being planewaves with infinite width in general. Consequently, conventional GMR CPA devices are highly sensitive to sizes of a device or incident beam.

A topological-junction GMR device can considerably alleviate or even completely remove these issues, as shown in Fig. 1b. We assume a junction of two topologically distinguished waveguide gratings with parameters *F*_L_ = 0.35, *a*_L_ = 560 nm, *F*_R_ = 0.45, and *a*_R_ = 540 nm with subscripts L and R denoting the left and right side of the junction, respectively. This parameter set is derived such that the two gratings about the junction have opposite Dirac-mass signs, which are determined by a formal mapping of the photonic wave equation onto the one-dimensional Dirac equation for relativistic elementary particles^16^.

In Fig. 1b, we assume the identical coherent Gaussian-beam incidences at wavelength 1493 nm. No in-plane leakage loss is observed and the guided-mode field is well confined within the incident beam region, implying better mode match. Subsequently, the absorptance is improved up to 80%. In Fig. 1c, we compare the phase-sensitive absorptance-modulation profiles of the conventional and topological-junction cases. We note that the proposed topological junction structure can enhance the absorptance peak up to almost 100%, as we will demonstrate in the next section.

**Figure 2:**
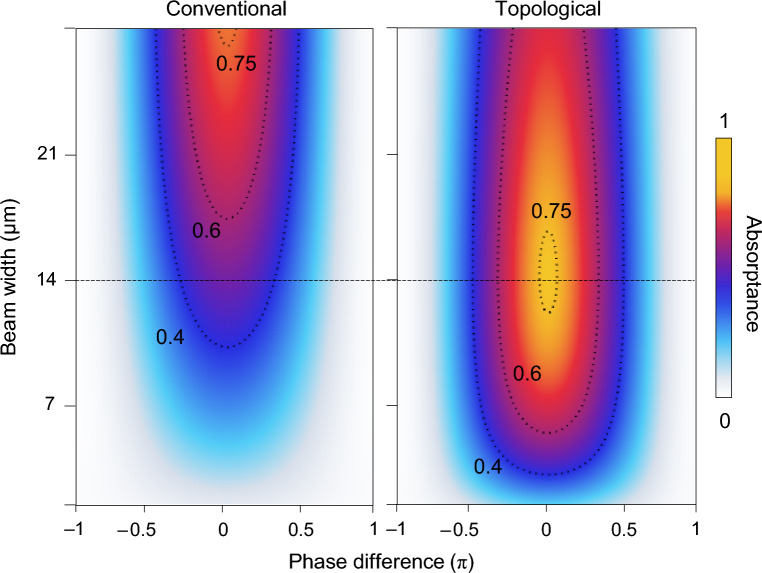
Impact of finite beam width on the performance. Incident-beam-width-dependent absorptance modulation profiles for the conventional and topological GMR CPA devices in Fig. 1. Footprint size of the devices is fixed at 50 μm.

We further investigate the finite beam-width effect on the coherent absorption properties for the conventional and topological junction structures. Figure 2 shows the phase-sensitive absorptance change for the Gaussian beam width varying from 2 to 28 μm. The absorptance is modulated by the interference while the phase difference between the incident beams are changing from − π to π. The modulation depth depends on the incident beam width. For the conventional GMR case, the modulation depth increases with the beam width (*W*) because the ideal point is obtained for the infinitely wide beam, i.e., planewave. In contrast, the topological junction case has its largest modulation-depth point at *W* = 14 μm for our particular design. This optimal beam-width condition is obtained when the incident beam profile best matches to the localized GMR state at the junction. The effect of finite beam size is closely related to angular tolerance of a resonance. In our case here, we identify an angular-tolerance width as angular full-width at half-maximum of a resonance peak. The calculated angular-tolerance widths for the conventional GMR at off-normal-incidence and normal-incidence conditions are 0.16° and 0.8°, respectively. These values are substantially smaller than the topological junction structure which has 1.6° for the angular-tolerance width. We also note in the small beam-width region below 14 μm that the large modulation depth domain is much more persistent for the topological junction case, which is highly desirable for compact device development.

### Topological mode-matching method

In analogy to the zero-energy state solution of the Dirac equation, a junction of two topologically distinguished lattices supports a leaky JR state^16,17^ at the center of the bandgap. In such structures, optical near-field and radiation patterns of this leaky JR state can be conveniently described, using the formal analogy of the photonic coupled-mode theory to the one-dimensional Dirac equation. The theory describes the transversal electric field for the JR state as a localized standing wave

1 $$ \psi_{{{\text{JR}}}} = u(z)(e^{iGx} + ie^{ - iGx} )f_{{{\text{JR}}}} (x), $$

where *u*(*z*) is the cross-sectional wave function of the guided mode, *G* = 2π/*a* is the grating vector, and *f*_JR_(*x*) = $\text{exp}[{\int }_{-x}^{x}-m\left({x}{\prime}\right)dx{\prime}]$ is the lateral envelope function of the JR state with *m* denoting the Dirac mass parameter. Consequently, Eq. (1) describes the localized state part *ψ*_JR_ of the total electric field, which is confined within the guided-mode envelope function *u*(*z*). Note that a π/2 phase difference between two standing guided modes *u*(*z*)*e*^*iGx*^ and *iu*(*z*)*e*^*–iGx*^ arises due to the phase delay from the Bragg reflection processes, respectively. As previously discussed in Ref.^18^, *f*_JR_(*x*) also describes the beam envelope of the coupled leakage-radiation distribution *f*_leak_(*x*), *i.e.*, *f*_leak_(*x*) = *f*_JR_(*x*). The relation between *f*_leak_(*x*) and Dirac-mass distribution *m*(*x*) can be alternatively expressed as

2 $$ m(x) = - \frac{1}{{f_{{{\text{leak}}}} }}\frac{{df_{{{\text{leak}}}} }}{dx}. $$

Equation (2) provides a convenient method for the complete mode match essential for the ideal CPA operation. An essential condition for the ideal CPA state is to have coherent incident beams configured such that they are the exact time-reversal of the leakage-radiation from the localized mode. This implies that *f*_leak_(*x*) must be matched to the incident beam profile for the ideal CPA state. In this perspective, a complete mode match can be obtained for a given arbitrary incident beam profile *f*_inc_(*x*) by configuring *m*(*x*) according to Eq. (2) with the mode-matching condition *f*_inc_(*x*) = *f*_leak_(*x*).

In experimental situations, obtaining the mode-matching condition *f*_inc_(*x*) = *f*_leak_(*x*) is possible in two opposite ways in general. One is to carefully configure *f*_inc_ by means of available beam-shaping methods. The other is to configure *f*_leak_ by means of nanophotonic structure optimizations. The latter is desirable in many practical situations, where *f*_inc_ is predetermined by the specific choice of sources and other required conditions. However, configuring a desired *f*_leak_ is basically nontrivial and involves tedious numerical optimizations that often do not ensure an ideal solution. The topological GMR CPA device with Eq. (2) takes a remarkable advantage within this context.

The mode-matching condition by means of Eq. (2) can be conveniently obtained by appropriately designing unit-cell geometry. For a rectangular grating-ridge unit cell of our interest here, the Dirac mass is determined primarily by fill-factor *F* of the grating as

3 $$ m(x) = \frac{{\pi n_{g} }}{{\lambda_{0} }}\Delta \varepsilon F[C_{1} \Delta \varepsilon \sin {\text{c}}^{2} (F) - C_{2} \sin {\text{c}}(2F)], $$

where sinc(*x*) = sinc(π*x*)/(π*x*) is the normalized sinc function, Δ*ε* = *n*_c_^2^–*n*_d_^2^ is the dielectric constant contrast in the grating layer, *n*_*g*_ is the group index of the guided mode, λ_0_ is the bandgap center wavelength, and *C*_1_ and *C*_2_ are dimensionless constants associated with the strength of the first- and second-order diffraction processes, respectively^17^. Therefore, we can systematically configure *f*_leak_(*x*) = *f*_inc_(*x*) for a given arbitrary *f*_inc_(*x*) by taking fill-factor distribution *F*(*x*) according to Eqs. (2) and (3).

We numerically demonstrate this topological mode-matching method for a most popular beam shape—Gaussian beam. For the trial case of Fig. 1b, we assume a piece-wise constant Dirac-mass distribution, *i.e.*, *m*(*x* < 0) = − *m*_0_ and *m*(*x* > 0) =  + *m*_0_, where *m*_0_ > 0. We refer to this case as “simple junction” hereafter. Thereby, the leakage radiation has a bidirectional decaying-exponential beam profile, which is not matched quite well to the Gaussian beam and results in the incomplete CPA at 80%. We provide an example that the lost 20% efficiency is almost completely recovered by means of the topological mode-matching method.

**Figure 3:**
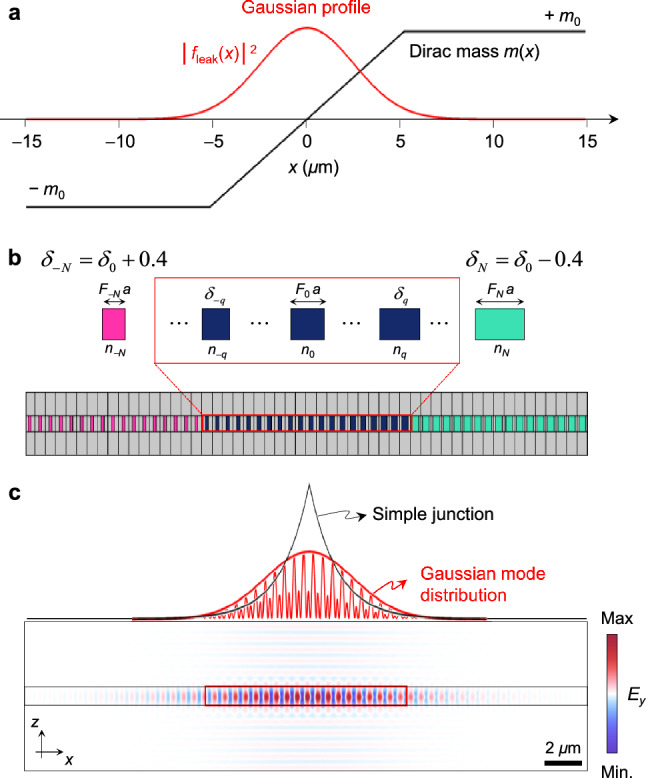
Topological mode-matching to a Gaussian beam. (**a**) Dirac mass *m*(*x*) distribution for the mode-matching condition in Eq. (2) with an exemplary Gaussian beam. (**b**) Schematic of a mode-matched topological junction structure. (**c**) Electric field *E*_*y*_ pattern of the JR state in the mode-matched structure in comparison with the simple junction structure.

Equation (2) implies a linear *m*(*x*) distribution for a Gaussian *f*_leak_ profile, as shown in Fig. 3a. We design each unit cell for such *m*(*x*) distribution by introducing a cell-by-cell parameter *δ*_*q*_ = *δ*_0_ − *q*Δ, where *q* is unit-cell index running over − *N* to + *N*, *δ*_0_ is a critical point for *m* = 0, and Δ is constant increment, as shown in Fig. 3b. For the sake of convenience in this proof-of-concept demonstration, parameter *δ*_*q*_ simultaneously tunes *n*_c_ and *F* of the *q*-th unit cell such that *n*_c*q*_ = *n*_d_ + *δ*_*q*_ and fill factor *F*_*q*_ = (*n*_avg_ − *n*_d_)/*δ*_*q*_ with *n*_avg_ = *F*_0_ *n*_c0_ + (1 − *F*_0_)*n*_d_. Equation (3) for Eq. (2) is conveniently mapped onto the unit cell geometry in this way.

The result is provided in Fig. 3c. The design parameters are *δ*_0_ = 1.035, Δ = 0.04, *N* = 10, *α* = 4.4 × 10^–3^, *n*_d_ = 2.45, *n*_avg_ = 2.864, *a* = 550 nm, and *d* = 500 nm. These parameters are optimized for a Gaussian JR-state envelope with beam diameter 9.4 μm. We show the mode-matched Gaussian envelope in comparison with the simple junction case where *m*(*x*) is piece-wise constant at − *m*_0_ for *x* < 0 and + *m*_0_ for *x* > 0, of which values correspond to the cell-by-cell parameter *δ*_0_ + 0.3 and *δ*_0_ − 0.3. The 2D field distribution of the JR state for the mode-matched junction is provided below.

**Figure 4:**
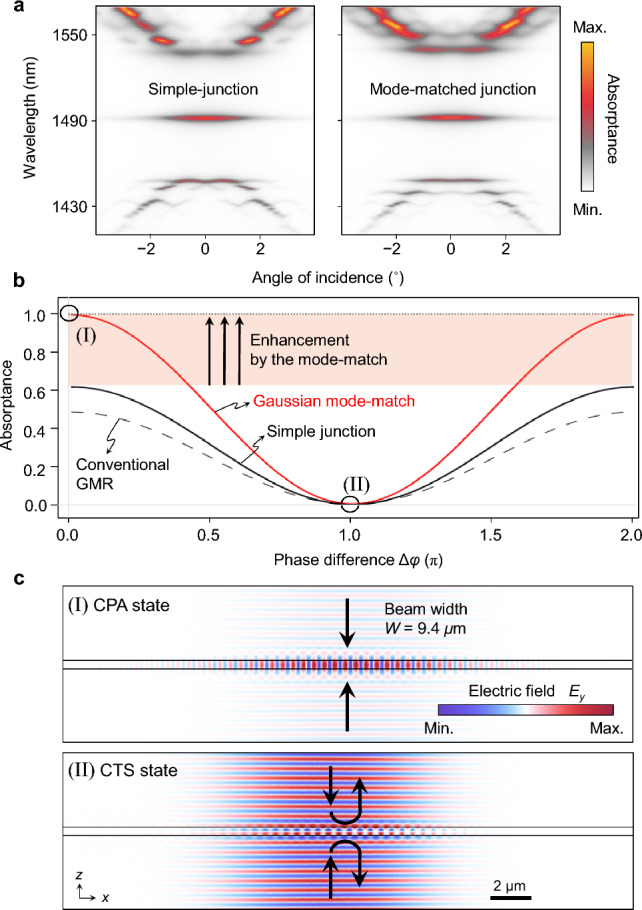
Almost ideal performance enabled by a mode-matched topological GMR CPA device. (**a**) Angle-dependent absorption spectra for the simple and mode-matched junction spectra. (**b**) Phase-sensitive absorptance-modulation profiles of mode-matched junction structure in comparison with the conventional GMR and simple-junction cases. We assume coherent light incidence of 9.4-μm-wide Gaussian beams. (**c**) Electric field patterns at the in-phase CPA (I) and out-of-phase CTS (II) states.

The resonance band structure and phase-sensitive control performance of the mode-matched junction structure is shown in Fig. 4 in comparison with the simple-junction case. In Fig. 4a, we show angle-dependent coherent-absorption spectra for simple junction and mode-matched junction. We clearly identify the JR-state resonance in the middle of the bandgap region for both cases. See Method section for details of simulation conditions. The phase-sensitive absorptance modulation profile under a Gaussian beam incidence is provided in Fig. 4b. The mode-matching results in a remarkable performance enhancement from those for the simple-junction JR-state and conventional GMR cases, where parameter *δ* for the conventional GMR case is constant at *δ*_0_ − 0.3. Note that the area illuminated by the incident beam profile *f*_inc_(*x*) and the linear *m*(*x*) distribution for a Gaussian *f*_*l*eak_(*x*) profile must be aligned, so that they are ideally mode-matched in the excitation area. At the in-phase excitation condition (Δ*φ* = 0) with 9.4-μm-wide Gaussian incident beams, the absorptance becomes almost perfect at *A* = 0.989 when compared to *A* = 0.616 and 0.473 for the simple-junction JR-state and conventional GMR cases, respectively. Importantly, such a high efficiency is obtained for a remarkably small footprint size at 30 μm. In this comparison, the peak absorptance for the simple junction structure can be slightly enhanced up to 63% by reducing the beam width down to 8.5 μm. Thereby, the case provided in Fig. 4b is a near-optimal condition for the simple junction structure.

The mode-matched JR-state device enables highly efficient optical modulation between the CPA and CTS states by tuning the incoming phase difference from 0 to π as indicated by I (Δ*φ* = 0) and II (Δ*φ* = π) in Fig. 4b. Corresponding field distributions are shown in Fig. 4c. At the CPA state, strong excitation of the mode-matched JR state and no out-going wave are clearly observable. At the CTS state in contrast, the JR state is not excited due to the complete destructive interference in the resonance mode while the complementary constructive interference happens in the out-going wave field, leading to the complete scattering with no absorption. Therefore, this device can function as a compact coherent perfect absorber without any significant performance degradation in the previous conventional approaches.

The remarkable enhancement of the performance by mode matching suggests that the device performance should be sensitive to spatial alignment of the incident beam position with the junction center. The sensitivity can be inferred by considering spatial correlation, i.e., normalized spatial overlap, between the incident beam profile and JR-state envelope. In the spatial-correlation approach, the alignment tolerance can be roughly determined in a similar sense to the Rayleigh criterion for diffraction-limited optical resolution. Accordingly, the lateral tolerance width Δ*x*_c_ for in-plane alignment must be half-width at half-maximum *W*_JR_/2 of the JR state envelope, *i.e.*, Δ*x*_c_ ≈ *W*_JR_/2. For out-of-plane alignment along the surface normal axis, the axial tolerance region Δ*z*_c_ must be depth of beam waist, which corresponds to the Rayleigh range in case of Gaussian beams, i.e., Δ*z*_c_ ≈ (π*λ*^−1^)*W*_JR_^2^. For ideal performance, the lateral and vertical alignment errors Δ*x* and Δ*z* should be substantially smaller than Δ*x*_c_ and Δ*z*_c_, respectively. In our simulated case for Fig. 4, Δ*x*_c_ ≈ 4.9 μm and Δ*z*_c_ ≈ 50 μm. This implies that sub-micron level (~ Δ*x*_c_/10) precision is required for lateral alignment while micron-scale (~ Δ*z*_c_/10) precision is enough for vertical alignment in order to obtain ideal performance.

## Discussion

In conclusion, we have proposed a topological-junction coherent perfect absorber taking advantages of small device footprint and persistently high performance. Characteristic field distributions of a leaky JR state at the junction lead to efficient mode-matching to incident Gaussian beams. On this basis, we numerically demonstrate 30-μm-wide CPA device with almost ideal interferometric control of absorption and scattering of coherent incident beams with 10-μm diameter. Such properties are hardly available with the conventional GMR structures. Therefore, our result strongly encourages further extensive follow-up study including higher-order JR states for complete two-dimensional confinement and subsequent adoptable mode matching as well as practical applications to low-power optical modulators, light-electricity transducers, coherence filters, sensors, and many others.

In particular, absolute absorptance values at the CPA and CTS states are crucial in practice. Considering the responses in Fig. 4b for example, the modulation depth in the transmitted coherent signal between the CPA and CTS states is 19.5 dB for the beam-shaped topological junction, which is remarkably higher than 4.1 dB for the simple-junction case and 2.7 dB for the conventional GMR case. If we further consider coherence filter applications, impact of such modulation depth contrast is also significant. Suppose a coherence filter system that transmits coherent signal while rejecting incoherent noises below a certain required level, let’s say 10% of the incident noise. For this, we have to set the operation condition at the CTS state so that the coherent signal absorption is minimal. The incoherent absorptance is simply given by 0.5*A*_max_, where *A*_max_ is the absorptance at the CPA state. We then estimate the minimal number *N*_d_ of devices in a serial connection and absolute efficiency *η* of the transmitted coherent signal. We obtain *N*_d_ = 4 and *η* = 93% for the beam-shaped topological junction whereas *N*_d_ = 10 and *η* = 84% for the conventional GMR performing the same incoherent noise rejection capability. Besides such efficiency-wise comparison, the topological junction device is highly desirable in consideration of robustness against structural imperfections and errors, which are unavoidable in experiments. Although we do not provide detailed analyses for our particular cases treated, it is previously known well from Ref.^16^ that spectral location and resonance strength of a topological-junction GMR are insensitive to random parametric errors in the structural parameters such as grating ridge width and period.

## Methods

The calculation domain for supercell structure which consists of multiple of unit-cell elements was covered with perfect matched layers (PML) with the scattering boundary condition. The phase-sensitive absorptance profiles were calculated by surface integration of total power dissipation density for the supercell computational domain. The mode-matched Gaussian JR-state in Fig. 3c was obtained using the time stationary eigenfrequency solver for the supercell with the PML boundary condition. In the calculation of the angle-dependent absorption spectra, we use the same supercell computational domain and apply the periodic boundary condition to the lateral boundaries of the supercell.

## Data Availability

All data needed to evaluate the conclusions during this study are present in this paper. Request for additional information should be addressed to J.W.Y. at yoonjw@hanyang.ac.kr.
